# Mixed Reality Prototype of Multimodal Screening for Early Detection of Cognitive Impairments in Older Adults: Protocol Development and Usability Study

**DOI:** 10.2196/39513

**Published:** 2022-10-14

**Authors:** Monica Christova, Robert Strohmaier, Bianca Fuchs-Neuhold, Bernhard Guggenberger, Brigitte Loder-Fink, Theresa Draxler, Christoph Palli, Helmut Simi, Sandra Schadenbauer, Alexander Nischelwitzer, Gerhard Sprung, René Pilz, Robert Darkow, Wolfgang Staubmann

**Affiliations:** 1 Institute of Physiotherapy University of Applied Sciences FH JOANNEUM Graz Austria; 2 Section of Physiology Otto Loewi Research Center Medical University of Graz Graz Austria; 3 Institute of Business Informatics and Data Science University of Applied Sciences FH JOANNEUM Graz Austria; 4 Institute of Dietetics and Nutrition University of Applied Sciences FH JOANNEUM Graz Austria; 5 Institute of Health and Tourism Management University of Applied Sciences FH JOANNEUM Bad Gleichenberg Austria; 6 Department of Orthopaedics and Trauma Medical University of Graz Graz Austria; 7 Institute of Occupational Therapy University of Applied Sciences FH JOANNEUM Graz Austria; 8 Institute of Health Care and Nursing University of Applied Sciences FH JOANNEUM Graz Austria; 9 Department of Psychiatry and Psychotherapeutic Medicine Medical University of Graz Graz Austria; 10 Institute of Logopedics University of Applied Sciences FH JOANNEUM Graz Austria

**Keywords:** augmented reality, virtual reality, multimodal screening, cognitive impairment, smart cognition, elderly, usability, dementia, aging, screening tool, digital health, digital health intervention

## Abstract

**Background:**

The early diagnosis of cognitive impairments is an important step in the adequate management of dementia. The project “Smart Cognition & Behaviour Screening powered by Augmented Reality” (SCOBES-AR) aims to develop a multimodal screening tool (MST) for the early detection of cognitive impairments using augmented and virtual reality. The first project phase selected validated assessments for combination with the MST and tested it in 300 healthy older adults.

**Objective:**

This study established a protocol for the implementation and usability of a mixed reality (MR)–enhanced multidisciplinary screening tool for the early detection of cognitive impairments in older adults. The developed MST will be partially enhanced by MR, which is a combination of augmented reality (AR) and virtual reality (VR). This MR-enhanced prototype of the screening tool (MR-MST) will be tested and compared to the previously developed MST. The usability of the prototype will also be examined.

**Methods:**

This single-center observational crossover design study screens 100 healthy participants (aged 60-75 years) for cognitive decline using a specially developed MST (assessment of cognitive functions, olfactory sensitivity, nutritional preferences, gait parameters, reaction times, and activities of daily living) and an MR-enhanced MST in which the assessments of cognitive functions, reaction time, activities of daily living, and gait will be performed using tailor-made software and AR and VR hardware. The results of the MR-enhanced MST will be compared to those without MR. The usability of the developed MR-enhanced MST will be tested on 10 investigators and 10 test participants using observed summative evaluation and the codiscovery method, and on 2 usability experts using the codiscovery and cognitive walkthrough methods.

**Results:**

This study was funded by the Austrian Research Promotion Agency (grant 866873) and received approval from the ethics committee of the Medical University of Graz. The MR-MST and the experimental protocol for this study were developed. All participants gave written informed consent. As of July 15, 2022, a total of 70 participants have been screened. Data analysis and dissemination are scheduled for completion by September 2023.

**Conclusions:**

The development and testing of the MR-MST is an important step toward the establishment of the best practice procedure for the implementation of AR and VR in the screening of cognitive declines in older adults. It will help improve our knowledge of the usability and applicability of the developed prototype and promote further advancement in AR and VR technologies to be used in therapeutic settings.

**International Registered Report Identifier (IRRID):**

DERR1-10.2196/39513

## Introduction

Human neurocognitive functions decline with advanced age, but this decline may often exceed physiological cognitive aging and progress to dementia. The prevalence of dementia worldwide is expected to almost double every 20 years, from 46.8 million in 2015 to 131.5 million in 2050 [1], which poses an increasing challenge for health and social systems. On the way from normal cognitive decline to dementia, mild cognitive impairment (MCI) is regarded as a decline in the ability to learn new information or recall stored information [2]. MCI was detected in 10%-15% of the population older than 65 years and is regarded as a “window” in which suitable interventions may delay progression to dementia [3]. The possibility of slowing dementia raises awareness of this global health problem in the scientific and health communities, which involve a large spectrum of clinicians, neuropsychologists, dietologists, speech therapists, physiotherapists, and occupational therapists. Combating dementia has become a public health priority. In accordance with the global action plan of the World Health Organization against dementia [4], international research has focused on the identification and reduction of risk factors, development of prevention and therapeutic interventions, and early detection of cognitive decline [5].

Because of the complexity of dementia diseases, predicting the course of MCI is not always successful. However, the efforts of clinicians and health providers are focused on screening, early diagnosis, and identification of risk factors. Very early diagnosis of MCI enables health care providers to manage these factors or ideally to decrease the potential progression to dementia. Notably, the early detection of MCI signs increases patient awareness and self-management and encourages participation in clinical studies or prevention programs. Although the diagnosis of early dementia initially causes uncertainty [6], people affected by this diagnosis and their family caregivers report benefits of early detection [7]. Healthy populations, and especially people 60 years or older, tend to show increased intention and willingness to be screened for cognitive decline [8-11].

Screenings and preventive interventions among older at-risk individuals that target several modifiable risk factors were effective [12,13]. Current clinical diagnostic procedures, such as liquor diagnostics and imaging techniques [14-18], are highly important but are not suitable for population-based early detection. Therefore, alternative screening tools that provide a low-threshold, safe, and valid way are needed to detect subtle cognitive changes in the earliest clinical phase. Notably, the manifestation of MCI includes cognitive, motor, sensory, and behavioral domains. These complex characteristics require a miscellaneous assessment approach.

The screening tools for cognitive impairments are controversial because of their limitations when they are performed using pen and paper [19]. For example, the written form does not allow direct digitalization for further and automatic processing, and these tests do not resemble real-life situations. Therefore, the performance of screening tests using augmented reality (AR) and virtual reality (VR) technologies facilitate data recording and processing while simultaneously enabling multisensory interaction of the subject with a real environment. AR superimposes 3D virtual objects into the real environment, typically using a tablet or a smartphone [20], but VR offers the user a multisensory experience of a simulated 3D virtual world via head-mounted displays, such as Oculus Quest screening tools [21]. For screening tests that aim for the early detection of cognitive decline, an integration of multiple domains with smart technology solutions must be developed and examined to prove their usability and validity.

The rationale for the Smart Cognition & Behaviour Screening powered by Augmented Reality (SCOBES-AR) project [22] is based on evidence-based predictors and protective factors for cognitive decline to establish a suitable assessment instrument that may be easily implemented in a nonclinical environment. Therefore, a combination of validated assessments of cognitive, motor, and behavioral functions was tested in the first phase of the project on 300 individuals aged 60-75 years. The multimodal screening tool (MST) includes assessments of olfactory functions [23], specificity of nutritional behavior [24], changes in gait pattern [25] and reaction time [26], cognitive functions [27], and instrumental activities of daily living [28]. As a next step, the developed MST will be enhanced with mixed reality (MR) technologies (AR and VR), which will require the establishment and testing of the developed prototype. Some of the assessments in the MR-enhanced screening tool will be performed using specially developed software and suitable AR and VR hardware, such as smartphones mounted in Haori headsets (AR) and Oculus Quest 2 (VR) headsets. This single-center observational crossover design study aims to establish a protocol for the implementation and usability of a prototype screening tool for the early detection of cognitive impairments in older adults using an MR environment.

## Methods

### Study Design, Participants, and Recruitment Procedure

This study developed a protocol for a single-center observational crossover design study. Male and female volunteers (N=100) aged 60 to 75 years were recruited from the pool of participants who had participated in the previous phase of the project. The volunteers were invited to a second visit at the Health Perception Lab, FH JOANNEUM University of Applied Sciences. Assuming that the effects were larger than the previous project phase, an effect size of 0.35 was selected. Therefore, with α=.05 and β=.95, a sample size of approximately 100 participants was considered sufficient for this study. Feasibility reasons and recruitment experience were also considered for the laboratory setting and geographical region. The recruitment and enrolment process for participants with stringent inclusion criteria (ie, age 60-75 years; absence of movement; and visual, auditory, neurocognitive, and psychiatric disorders) lasted from March 2022 to April 2022. For recruitment, participants were contacted by email or telephone to obtain consent and make preliminary agreements for participation. The study procedure, including the application of the respective information and technology devices, was presented to the volunteers. Participants were excluded from the study if they developed 1 or more of the conditions described in Textbox 1.

**Study exclusion criteria.**

- Clinical diagnosis of mild cognitive impairment or dementia
- Clinical diagnosis of mental illness (eg, depression, psychosis)
- Reduced mobility (eg, walking aid, wheelchair)
- Aided hearing or visual impairment
- Participation in any other cognitive training study within the last 6 months
- Present guardianship according to the provisions of the Austrian adult protection law

All selected volunteers are tested once with the MST and once with the MR-MST in a randomized order. An independent investigator executed the randomization plan using a computer-generated random numbers table.

### Ethics Approval

The study is performed in accordance with the Helsinki Declaration and its later amendments, and all procedures involving human participants have been approved and accepted by the local ethics committee of the Medical University of Graz (EC No. 32016 ex 19/20, 14-17 2019). The participants are informed in detail verbally and in writing about the procedure and aim of the study. Participation in the study is ensured by signing a written informed consent form. As compensation for the time spent in the study, participants receive a voucher (equivalent to €15 or US $14.80). Access to the generated data is restricted to the immediate research team, and only coded data stored on a secure internal server of the FH JOANNEUM, University of Applied Sciences, are used for analyses.

### Indemnity

This study is not an intervention study and poses a low risk to participants. For some attendees, the test results may indicate a deviation in cognitive abilities, which suggests early cognitive decline. When this occurs, we provide a comprehensive medical consultation with the study’s clinical investigator to ensure further medical care and possibly determine a further diagnostic plan. Participants may report slight fatigue when participating in the different assessments. A short preventive break is taken after approximately half of the testing procedure.

Part of the screening procedure involves wearing a head-mounted display with VR, which may cause cybersickness [29]. Cybersickness is triggered by visual stimuli during exposure to the virtual environment and shares similar symptoms with motion sickness, including nausea, sweating, dizziness, and fatigue. To minimize the risk of cybersickness, the participants undergo screening in a static (sitting or standing) position. The participants could withdraw from the screening in case of cybersickness. Except for these considerations, no health risks or side effects are expected for the participants.

### Study Procedure

**Figure 1:**
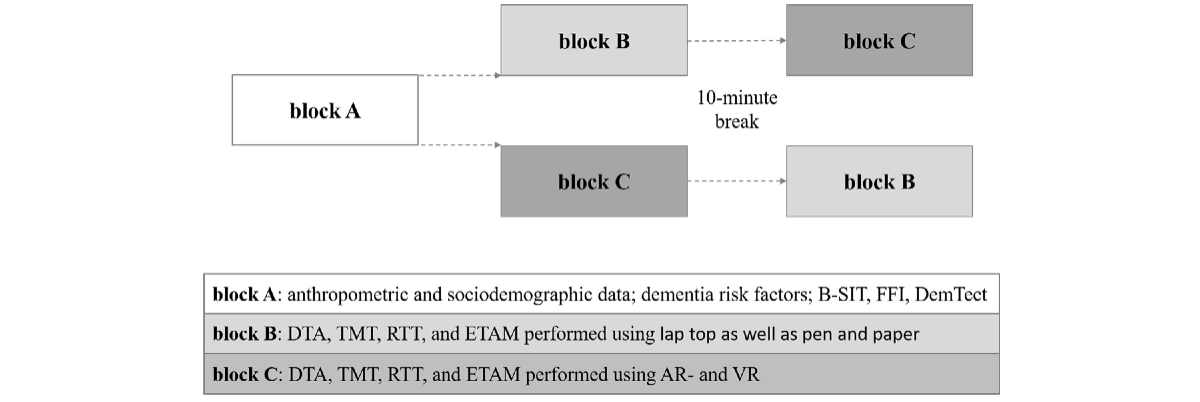
Experimental design. AR: augmented reality; B-SIT: Brief Smell Identification Test; DemTect: Dementia Detection Test, DTA: Dual-Task Assessment; ETAM: Erlangen Test of Activities of Daily Living in Persons With Mild Dementia or Mild Cognitive Impairment; FFI: Food Frequency Index; RTT: Reaction Time Test; TMT: Trail-Making Test; VR: virtual reality.

At the beginning of the study, the investigator clarifies in detail the screening procedure and COVID-19–related hygiene measures and obtains signed written informed consent from the volunteers. The screening procedure for each participant consists of 3 blocks (see Figure 1). Anthropometric, sociodemographic, and risk factor data are collected in the first block (A). The Brief Smell Identification Test (B-SIT) [23], Food Frequency Index (FFI) [30], and Dementia Detection Test (DemTect) are also performed in block A using a laptop computer as well as pen and paper. The Dual-Task Assessment (DTA) [25,31,32], Trail-Making Test (TMT) [33], Reaction Time Test “Match 4 Point Test” [34], and Erlangen Test of Activities of Daily Living in Persons With Mild Dementia or Mild Cognitive Impairment (ETAM; parts transport and finances) [34] are performed in the second block (block B) using software as well as pen and paper. The same assessments from block B are performed in the third block (block C) using MR technologies, tailor-made MR software, and suitable AR and VR hardware. Prior randomization determines the order in which participants perform blocks B and C.

The entire testing procedure lasts approximately 130 minutes, including a 10-minute break between blocks B and C. Trained project staff conduct the study. A medical doctor acting as the clinical investigator is available to the participants for consultation to answer questions related to their cognitive health.

### Methods of Measurement in the MST

The selection of the assessments included in this screening tool was based on the results of a systematic review process, focus groups, and an observational study (unpublished data), which were performed in the first phase of the SCOBES-AR project. The assessments were evaluated based on predefined criteria related to their quality and applicability for the purpose of the project. Assessments from cognitive, motor, sensory, and behavioral domains with proven validity and reliability were chosen for this study.

#### B-SIT Procedure

A decrease in olfactory perception is a predictive factor for the development of neurocognitive impairment [35,36]. The B-SIT [23] is a screening test for odor recognition, and it is a short version of the widely distributed University of Pennsylvania Smell Identification Test. Different versions of the B-SIT have shown validation sensitivity/specificity ratios between 83.1%/79.5% and 96.5%/51.8% [37]. For the test, 12 different microcapsulated fragrances were painted over with a pencil for release in a standardized quantity (scratch-and-sniff test). The person undergoing the test must detect the released odor and extract it from a 4-step multiple choice question. A total odor score results from the number of correctly identified fragrances, with higher values indicating better olfactory performance (>9 detected fragrances).

#### FFI Determination

A healthy diet helps reduce the risk of cognitive impairment and dementia [38]. Observational studies showed that omega-3 polyunsaturated fatty acids and vitamins, such as the B-complex (vitamins B6, B12, and folate), antioxidants (vitamins A, C, and E), and vitamin D, have a protective effect against brain aging [39]. Therefore, the assessment of nutrition quality and patterns shows a much broader picture of dietary behavior than individual nutrients [24,40]. The FFI is a 10-item index for the assessment of dietary behavior in Austrian adults. The index is based on the Austrian recommendations for food intake [30]. For each food group, 8 frequency expressions are available, and a maximum of 7 points may be achieved. Nutritional behavior is classified as poor (FFI<32), moderate (FFI=32-34), good (FFI=35-39), and very good (FFI>39). The FFI was validated with biochemical indicators. Higher FFI scores were associated with mean nutrient intakes closer to current nutrient-based dietary guidelines (ie, lower intakes of fat and cholesterol and higher intakes of total carbohydrate, fiber, and micronutrients). Significant correlations between FFI levels and the intake of saturated fat (*P*=.004), vitamin C (*P*=.02), and vitamin B6 (*P*=.04) were reported [30]. The test is implemented using a laptop computer.

#### DemTect Screening

The DemTect was designed as a highly sensitive psychometric screening process to identify patients with MCI and patients with dementia in the early stages of the disease. Compared to the known Minimental State Examination [41], classification rates of the DemTect were superior for the MCI and dementia groups, with high sensitivities of 80% and 100%, respectively, especially in incipient and only slightly advanced cognitive disorders [42,43]. The DemTect includes 5 short easy-to-administer cognitive tasks that are sensitive for the early diagnosis of dementia (immediate recall of word lists, number transcoding, semantic word fluency task, and digit span reverse delayed recall of word lists). The test is implemented using a laptop computer. A score is determined for each task of the test, which is then converted to a test score using a conversion table. The test values are ultimately summed and evaluated using a scale that indicates the presence and severity of cognitive impairment.

#### DTA Procedure

Walking while performing a secondary cognitive task (dual-task paradigm) has become a classic way to assess the relationship between cognition and gait. Performance in a dual task has been associated with global cognitive function in older individuals with preserved cognition [44,45] and with MCIs [46-48], and it significantly correlates with the progression of dementia. DTA [25,31] measures the influence of the cognitive task on the gait pattern. DTA has demonstrated high test-retest reliability and strong criterion-related validity in older adults with MCI and high sensitivity (effect size=0.9) in detecting MCI [49]. For this study, a walking distance of 10 meters and an additional 2 meters for acceleration and deceleration were chosen. Participants in block B are asked to walk the distance at a self-paced speed (single task). They are then asked to walk the 10-meter track and count backward loudly from 100 in increments of 3 (ie, dual task). Spatial and temporal gait parameters are measured using a stopwatch and a mechanical counter. Based on the walked distance, the needed time, performed steps, and spatial and temporal gait parameters are calculated (eg, walking speed, cadence, and stride length). From the single- and dual-task results, a percentage difference, which is defined as cognitive cost, is calculated [50]. DTA in block C is performed using a Google Pixel 6 smartphone (Google) mounted in a Haori headset (Shenzen Haori Technology Co, Ltd). Tailored AR software provides instructions to the participants and measures spatial and temporal gait parameters. Gait parameters are measured by detecting the walked distance and counting the time using Google ARCore [51]. The unity asset called Android StepCounter enables access to smartphone sensors and software algorithms to detect steps. The procedure is identical to that used for block B.

#### TMT Procedure

**Figure 2:**
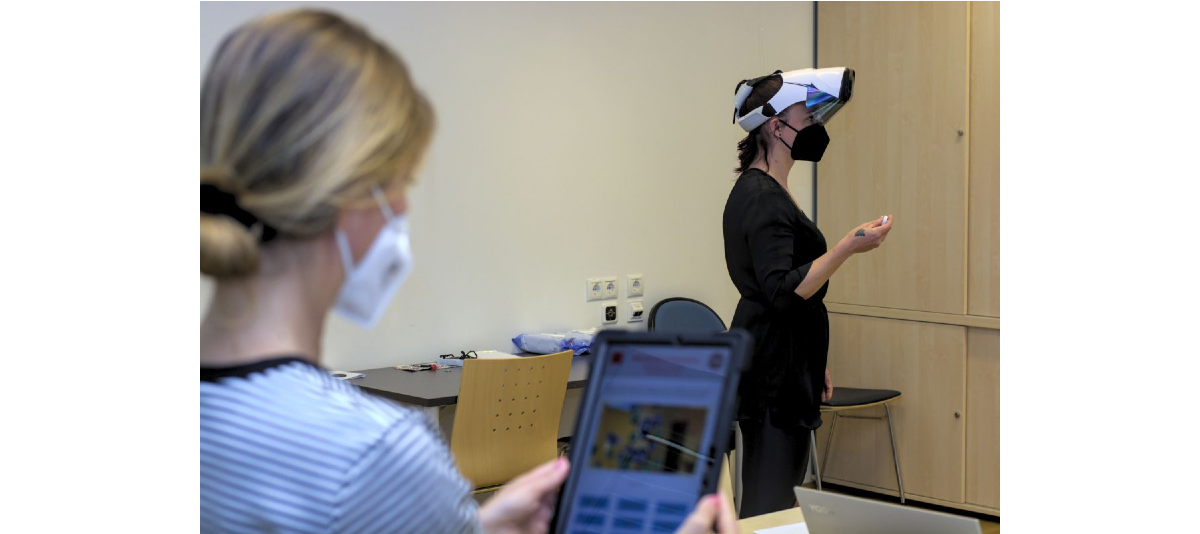
Trail-Making Test using Haori headset.

TMT may be used for the identification of attention disorders and executive dysfunctions [52], and screening for cognitive dysfunction [53]. Discrimination validity of TMT in dementia versus nondementia participants indicated sensitivity/specificity ratios of 75%/62.6% (TMT-A) and 62.5%/76.4% (TMT-B) [54]. TMT evaluates cognitive abilities and consists of two parts: (1) connecting numbers and (2) connecting numbers and letters in ascending order. The test is completed using pen and paper in block B. The MR-enhanced TMT in block C is performed using a Google Pixel 6 smartphone mounted in a Haori headset to enrich the real world with virtual objects (Figure 2). The virtual objects are spheres, labeled with numbers and letters. These spheres are anchored in real space. Participants select the spheres in the correct sequence by looking at them and pressing a real button with their hand. The completion times in seconds are separately recorded for both test parts (numbers, and numbers and letters) in blocks B and C. A maximum of 100 seconds is provided for the test part “numbers” and 300 seconds for the test part “numbers and letters.”

#### Reaction Time Test: Match 4 Point Test

Studies on reaction time demonstrated that a slowing of the reaction time and an increasing intraindividual performance variability represented an important marker for the presence of dementia and MCI [55-57]. The Match 4 Point Test evaluates the complex reactions (eye-hand, eye-leg, and eye-hand-leg reactions) in response to visual signals displayed in a randomized order. Studies on the reliability and validity of the test demonstrated good test-retest reliability (*r*=0.82) and an acceptable correlation for convergent validity (*r*=0.61) compared to the results of the visual reaction test S1 by Schuhfried [58,59]. The test in block B is performed using the Talent Diagnose System (Werthner Sport Consulting) [25]. The Talent Diagnose System implements 3 contact plates (2 for the hands and 1 ground contact plate marked for the right and left foot), placed on a table (hands) and under a table (feet). A laptop computer displays 20 visual signals. The test participant is in a standing position and is instructed to touch the relevant contact plates as fast as possible using the left/right hand/foot in response to the given visual signal. The MR-enhanced “Match 4 Point Test” is performed in block C using a Google Pixel 6 smartphone by running a tailor-made mobile app and using 4 Flic 2 (Shortcut Labs) buttons, which are connected with the smartphone via Bluetooth. This app displays visual signals in the form of black and white shapes. Depending on the color and position of the signal, participants push the Flic 2 buttons with their left/right hand/foot. The time taken to complete the task is recorded.

#### ETAM Procedure

Activities of daily living are restricted in individuals with mild cognitive declines [28,60,61] compared to healthy controls. ETAM is a valid and reliable instrument for assessing activities of daily living in persons with MCI or mild dementia [62]. ETAM provides satisfactory discrimination between healthy individuals and persons with MCI or mild dementia [28,34] with test-retest reliability (*r*=0.78) and interrater reliability (*r*=0.97) [34]. The ETAM consists of six items that cover the following domains according to the Classification of Functioning, Disability and Health: communication, mobility, self-care, domestic life, and major life areas (specifically, the economic life subcategory referred to as finances). The items mobility and finances are implemented using pen and paper in block B and VR in block C.

**Figure 3:**
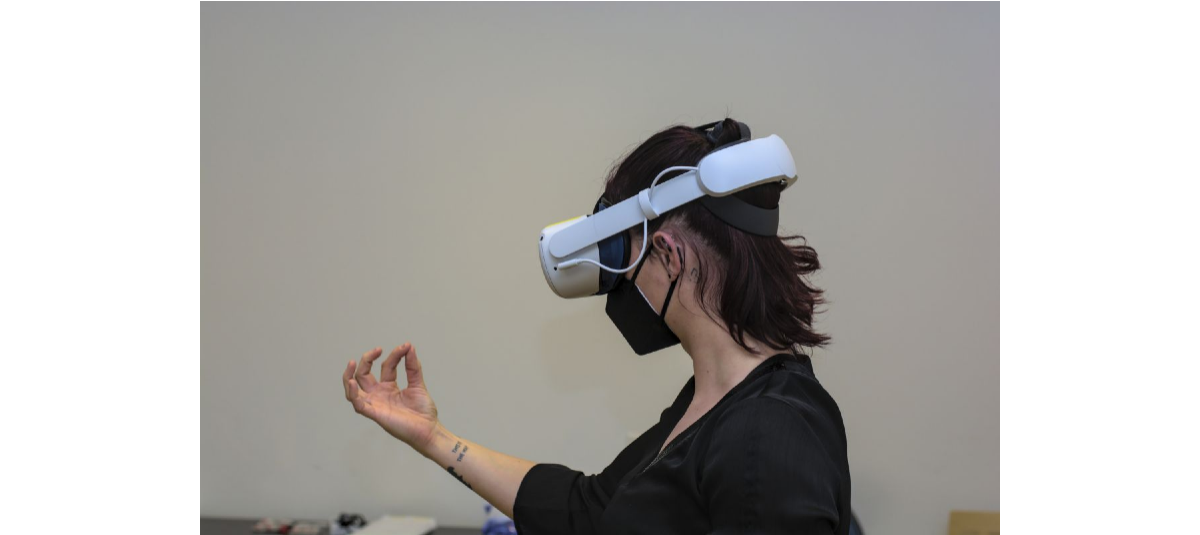
Erlangen Test of Activities of Daily Living in Persons With Mild Dementia or Mild Cognitive Impairment using Oculus Quest 2 virtual reality headset.

The task “traffic” (mobility) is related to understanding basic rules in road traffic situations and is tested based on 6 everyday traffic scenarios (eg, traffic lights). Participants in block B are shown photographs of 6 different traffic situations and asked to proceed and behave accordingly. The task “finances” (economic life) includes skills to compare offers, sum amounts, and count money. Participants receive three advertising leaflets and a stack of coins and are asked to buy the following grocery items: 1 pack of butter, 1 liter of milk, and 1 bread roll. They are supposed to pick the cheapest offer for butter from the 3 leaflets and calculate the total amount they need for all 3 products. Finally, they should take the necessary amount from the stack of coins in front of them. Performance in both tasks is evaluated using the ETAM scoring system. The same tasks are performed in block C using an Oculus Quest 2 VR headset (Figure 3). For the traffic task, participants are virtually “placed” in a spherical 360° video-recorded street environment and asked to navigate from a starting point to the pharmacy. Their behavior with respect to the traffic situations is documented automatically. Navigation within the 360° videos is performed in a standing position, using rotations, tilting of the body, and head and hand gestures. For the finances task, the participants are “placed” in a 3D grocery store. They interact with the virtual environment using hand gestures. Similar to the pen and paper version of ETAM, participants need to recognize the correct goods, calculate spending, and handle money in the virtual shop. The performance is scored based on the ETAM scoring system and automatically recorded.

### Software Development of the MR Screening Tool Prototype

**Figure 4:**
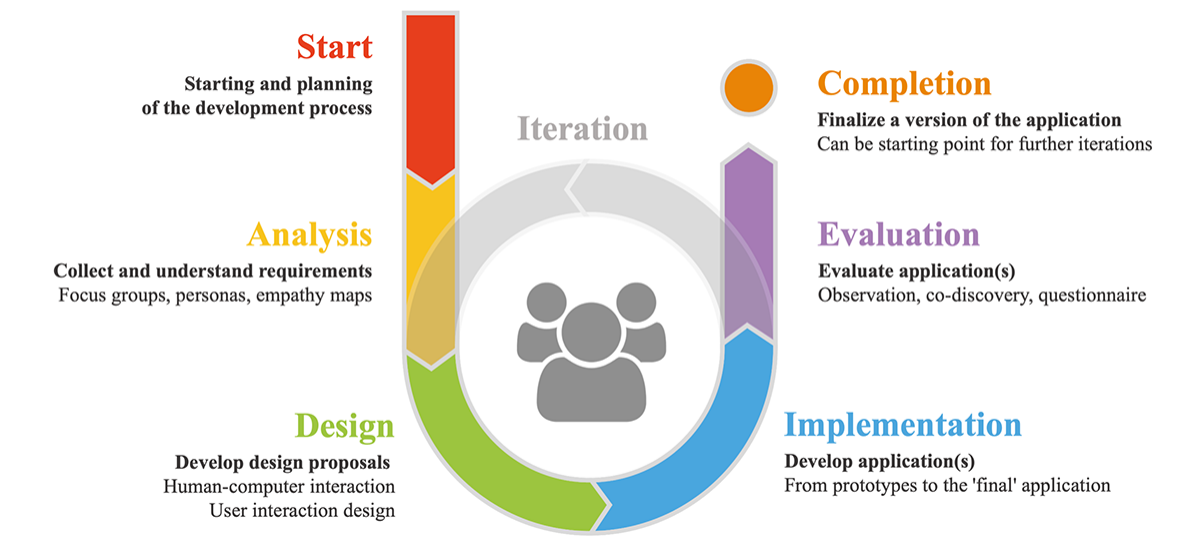
User-centered design process of prototype development.

The MR-enhanced MST is implemented using information and communication technologies. After analyses of the available hardware and software technologies and interfaces, technical and data management concepts were established for the project domain of SCOBES-AR. Selected tests from the screening tool were implemented using a Google Pixel 6 smartphone (Google) mounted in a Haori headset, an Oculus Quest 2 VR headset (Facebook Technologies, LLC), Flic 2 buttons, and tablets or computers for monitoring and annotation. Multiple software prototypes were developed by a team of digital media specialists and customized to meet the users’ needs when operating the devices. Therefore, we adapted a user-centered design process [63,64] and applied it iteratively in the software development. The process of software planning, development, design, analysis, evaluation, and final application is illustrated in Figure 4.

To obtain a deep understanding of the users of the screening tool prototype, target groups of older adults and therapists were identified at the start of the study, and the context of use was clarified [65]. The older adults and therapists separately participated in 2 focus groups [66], in which the study purpose and the user’s risks, problems, and benefits were discussed in detail. Based on the gathered information, personas [63,65,66] and empathy maps [65] were generated. These tools provided important feedback for the development process. Screenings of the test battery were conducted in the design phase for transformation into software applications. Selection and transformation of the screenings occurred in cooperation with health professionals with the help of early functional prototypes. The key features of the software for MR-MST were defined in group discussions in the focus groups. The implementation of the software prototypes occurred in the cross-platform development environment of Unity (Unity Software Inc) [67], assisted by plugins for Google ARCore [68] and Oculus Quest 2 [69,70]. The software prototype itself was designed as multiple mobile apps that could be installed on the target platforms, namely, smartphones with an Android operating system [71] and Oculus Quest 2 [70].

### Usability of the MR-Enhanced Screening Tool Prototype

The usability evaluation is performed to estimate the ease, efficiency, and satisfaction of using the prototype. The usability of the MR-enhanced screening tool prototype is evaluated by 2 testing groups implementing different methods. The usability tests focused on the assessments included in block C (MR-MST).

The first testing group includes 10 investigators and 10 participants in the screening procedure, acting as users (therapist/client). They evaluate the usability of the overall process, including all software applications and hardware devices used in block C. Traditionally conducted subscreenings will be assessed via observed summative evaluation [63,72] using the codiscovery [63] method. Using this method, both users act as they would in the performance of conventional screening without any interruption by the test supervisor during the test procedure. No additional tasks will be presented to them. These observations may be seen as unmoderated [73] usability tests, where usual screening occurs to provide qualitative (eg, behavior analysis afterward) and quantitative (eg, task completion times) feedback. The test supervisor will perform follow-up questionnaires after each observation to gain deeper insights into the interaction with the user interface. The questions will be personalized for each target group (ie, investigator and participant). A total of 10 screenings will then be observed with multiple video cameras, microphones, and screen recording apps for tablets. Afterward, usability experts will analyze the observations and questionnaires. This usability evaluation should deliver findings related to the organization of the complete process in block C and the user’s interaction with all software components.

The second testing group will include 2 usability experts, who will gain deeper insights into the usability of the complete system in the form of codiscovery using the cognitive walkthrough method [74,75]. During a cognitive walkthrough usability analysis, experts will look at action sequences using a product (eg, hardware, software, or both) through the user’s eyes. They will try to accomplish common action sequences that the target group will perform and detect as many usability problems as possible.

### Data Management

This study is performed by a multidisciplinary team of psychologists, speech therapists, occupational therapists and physiotherapists, dieticians, nursing scientists, information managers, software engineers, and biomedical analysts. All data will be saved and processed in a pseudonymized form using automatically generated alphanumeric codes. The list for identification is securely stored in a password-protected document in the study center and may be accessed only by the principal investigators. When published, all data will be anonymized. The collection, processing, and storage of the data follows the legal provisions of the General Data Protection Regulation (Regulation EU 2016/679) and requires signed informed consent. The data gathered from the test methodology (questionnaires) are collected and stored on a telehealth platform [76] provided by the Austrian Institute of Technology GmbH (subcontractor). The telehealth platform provides standardized data collection and interfaces based on Fast Health Interoperability Resources questionnaires [77] and on user interfaces for the medical personnel within the SCOBES-AR project.

### Outcome Parameters and Data Analysis

Statistical analyses will be performed to identify possible correlations between the different components of the MST and MR-enhanced MST. Spearman rank correlations (Spearman ρ) or product-moment correlations (Pearson correlations) will be calculated, depending on the distribution of the collected data. Multiple regression analyses will be conducted for cases of strong effects in the correlation between the MST and MR-enhanced MST. Descriptive statistics of the data will be presented as means and SDs. The level of significance is set at α=.05. The statistical data will be evaluated using SPSS (IBM Corp).

For usability assessment, usability experts will collect and analyze data from the video observations and questionnaires. The video observations will be evaluated using “Eisenhower's Urgent/Important Principle” [78]**,** in which items are classified as “not urgent” to “urgent” and as “not important” to “important.” The “urgent” and “important” items indicate higher needs of improvement. The questionnaires addressing the personal experiences with the screening procedure are based on a 5-point Likert scale, where 1 indicates “very good usability.”

## Results

This study was funded by the Austrian Research Promotion Agency (FFG, grant 866873) and was approved by the ethics committee of the Medical University of Graz, Austria. The funding body and funding source are not involved in performing the research and do not play any role in the execution of the study, data analyses, or result interpretation. All described procedures related to the equipment and software development, preliminary tests, study organization, and recruitment plans were completed. The investigators involved in the test procedure were trained to use the MR prototype and perform the entire screening procedure in joint and separate sessions. As of July 15, 2022, a total of 70 participants have been screened. Because this study describes the protocol for the implementation of the MR-enhanced multimodal screening tool, no results are currently available. Data analyses and result dissemination will be completed by September 2023.

## Discussion

### Overview

The increasing availability of AR and VR technologies provides an opportunity to translate available screening tools from the laboratory to the real world. This translation is challenging because of the current limited evidence in clinical research. Therefore, the development and testing of prototypes and research protocols are important steps in the successful application of these novel technologies in health care practice. This study described the development process and research protocol for the implementation and usability of an MR-enhanced MST for the early detection of cognitive decline in older adults.

Selection of the single assessments included in this MST was based on their validity and reliability in detecting cognitive changes in healthy older adults and individuals with diagnosed cognitive impairments. The screening tool was tested in the previous phase of the SCOBES-AR project. Some of these assessments are performed for the first time in this way using MR technologies, which require integrated multidisciplinary knowledge and the development and testing of several software models.

The user-centered approach for the development of the software prototype was based on the collaboration between engineers (digital media specialists, information and communication specialists, and software developers) and end users (therapists and researchers with different expertise areas as well as older adults). Common challenges during the software design process were related to appropriate implementation of the different assessments with the MR technologies, documentation of results, and provision of guidelines for the therapists on how to use the different software and hardware products. The extensive exchange between engineers and users enabled the development of easy-to-use AR and VR software with reliable technical quality and attractive designs. This exchange enables a smooth transition from technical to clinical research settings, where the developed prototype will be applied for screening older adult test participants and further evaluated.

### Strengths and Limitations

We designed this study to compare the results of the MST (block B) to those of the MR-MST (block C). The performance in the first block may facilitate the performance in the second block. To minimize the “learning effect,” these blocks were randomized among participants. As the entire screening procedure has a duration of approximately 130 minutes, participants may experience cognitive fatigue, which could influence the results of the tests. The effect of possible cognitive fatigue was minimized by providing a resting break between the blocks and frequently obtaining feedback from participants. The order of assessments in blocks B and C was selected to ensure maximum convenience and time efficiency when changing from assessments performed using AR to those performed using VR in block C.

Another limitation of this study protocol is related to the fact that the experiments are performed at different times of the day (eg, in the morning and afternoon) and by different investigators. To reduce the impact of circadian rhythms on cognitive performance, we avoided testing in the early morning and late afternoon. To ensure standard testing conditions, all involved investigators obtained the same training using the AR and VR technologies and performed the screening procedure.

One strength of this study is that the assessments performed with VR included familiar situations and daily life activities (eg, crossing a street, shopping in a grocery store) in which immersion into the virtual world created a cognitive experience that aligned with real-life demands. The MR-enhanced assessments were designed to resemble the assessments performed with pen and paper as much as possible. We are aware that the results of MR-enhanced MST may not be directly comparable to those of the MST because of the specificity of the AR and VR equipment and environment. These findings will shed more light on the extent, art, and origin of possible differences, which will enable further adaptations and optimizations of the prototype.

Usability is another important consideration in establishing screening tools involving novel technologies. Therefore, this study describes the usability evaluation of the developed MR prototype. Feedback will be collected from the users (investigators and clients) via observations (video observations of the screening procedure) and questionnaires. Usability experts will focus on the users’ cognitive activities related to their specific goals and understanding when executing screening using the MR prototype. This approach should reveal possible limitations and user disadvantages. Notably, outlining the particular needs of improvement will encourage further optimization to enable further application of the MR-enhanced screening tool in therapeutic settings.

### Conclusions

The development and application of the described MR-enhanced prototype for the screening of early cognitive decline in older adults is an innovative and demanding process. Establishment of the respective methodological procedures is a saliant step toward identifying the best practices. The results of the study will provide more information on the usability, utility, and applicability of AR- and VR-enhanced screening tools, which may encourage further development of these models and their use in therapeutic settings.

## Abbreviations

AR: augmented reality
B-SIT: Brief Smell Identification Test
DemTect: Dementia Detection Test
DTA: Dual-Task Assessment
ETAM: Erlangen Test of Activities of Daily Living in Persons with Mild Dementia or Mild Cognitive Impairment
FFI: Food Frequency Index
MCI: mild cognitive impairment
MR: mixed reality
MST: multimodal screening tool
SCOBES AR: Smart Cognition & Behaviour Screening powered by Augmented Reality
TMT: Trail-Making Test
VR: virtual reality

## Data Availability

The data sets that will be generated and analyzed during this study will not be publicly available because the generated experimental data are only usable in the given context of the project and its aims, but they are available from the corresponding author upon reasonable request.
